# Incidence of severe sepsis and septic shock in german intensive care units - the insep study

**DOI:** 10.1186/2197-425X-3-S1-A223

**Published:** 2015-10-01

**Authors:** G Marx

**Affiliations:** Intensive Care Medicine, Universitätsklinikum Aachen, Aachen, Germany

## Intr

Sepsis is a frequent syndrome associated with high morbidity, mortality and long-term disability. Valid epidemiological data are important to monitor the burden for the health care system and to allocate finances to patient care and research.

## Objectives

Aims of the study were to estimate the incidence of severe sepsis and septic shock in German ICUs, and to establish a method allowing repeated prospective investigations of sepsis epidemiology.

## Methods

INSEP was designed as a prospective multicentre epidemiological longitudinal observational study. All patients treated on the ICU on Nov. 4, 2013 and all patients admitted to a participating ICU between Nov. 4, 2013 and Dec. 1, 2013 were included into the study and followed up for the occurrence of severe sepsis or septic shock during their whole ICU stay.

## Results

A total of 11.883 Patients were included in the study in 129 ICUs at 95 hospitals of different specialization, type and size in Germany. 1503 (12.6%) patients had severe sepsis / septic shock. 643 (42.7%) of infections causing sepsis were community-acquired and led to the hospitalization of patients. 860 (57.2%) infections were of nosocomial origin. The most frequent sites of infections were the lower respiratory tract (n = 700, 46.6%), the abdomen including the gastrointestinal tract (n = 431, 28.7%), and the urogenital tract (n= 190, 12.6%). An incidence density of sepsis of 11.76 [95%-CI: 10.62-12.99] per 1000 ICU-days was found. The point prevalence was 17.9% [95%-CI: 16.3-19.7]in the whole study population. ICU mortality related to severe sepsis / septic shock was 34.3%, approximately 5.5 times higher than in patients without severe sepsis / septic shock (6.0%).

## Conclusions

Severe sepsis and septic shock continue to be a frequent syndrome with a high mortality rate. The risk of developing septic shock increased with the number of SIRS criteria fulfilled. Multiorgan dysfunction syndrome occured frequently in patients with severe sepsis /septic shock. The high rate of nosocomial infections as a cause of sepsis demands further investigation. The presented kind of study is a pragmatic, affordable and feasible tool in the investigation of sepsis epidemiology.

## Grant Acknowledgment

The study was carried out by the German Competence Network Sepsis (SepNet) without financial funding. We thank all contributors for their engagement.
